# Macro Risk: A Versatile and Universal Strategy for Measuring the Overall Safety of Hazardous Industrial Installations in China

**DOI:** 10.3390/ijerph16101680

**Published:** 2019-05-14

**Authors:** Guantao Wang, Jingjing Pei

**Affiliations:** School of Engineering and Technology, China University of Geosciences, Beijing, Beijing 100083, China; wangtgt@cugb.edu.cn

**Keywords:** macro risk, calculation method, safety supervision, hazardous installations, loss equivalent

## Abstract

In this work, macro risk is used to describe the overall safety of a group of hazards that are congeneric in a certain area, which can effectively help safety supervisors with goal setting and decision-making in China. To demonstrate this, the article proposes a calculation method to quantitatively study the macro risk of hazardous industrial installations. The method simultaneously considers the probability and consequences of accidents as the two core elements of risk, and the consequences cover losses with various dimensions. Assisted by related probability theory and binomial distribution, we analyzed historical accident statistics in detail to reveal hidden laws. To explore how to normalize the dimension of varied losses, the number of person-years was introduced as a loss equivalent to set up a method of conversion between loss of life and economic loss. The calculation method, which manifests a versatile and universal strategy of macro risk, was thus established. The value of the macro risk obtained possesses chronergy. Based on chronergy, two applications in China are further discussed, indicating this method is indeed feasible and practical for safety supervision. Specifically, it can help reasonably allocate regulatory resources by comparing macro risks of the same types of installations in various jurisdictions. In addition, it is conducive to a scientific determination of regulatory direction through the comparison of macro risks of various types of installations in the same jurisdiction.

## 1. Introduction

Risk management is an active systematic approach [1] to effectively preventing accidents. In the past three decades, risk management has emerged as a necessary scientific tool that plays a significant role in the safety management/supervision of enterprises and governments at all levels [2]. There are three main items in the risk management framework that dominate the operations of an entire system: risk analysis, risk evaluation, and risk control [3]. Among them, risk analysis is a process of systematically identifying hazards and analyzing the likelihood and the consequences of related accidents that could potentially occur. The process of risk evaluation, which is designed to judge whether the obtained risk has reached an acceptable level [3], is based on the results of risk analysis. To better serve decision-making regarding risk (risk control), neither the analytical process nor its results should be considered in isolation [4]. In this context, risk assessment aims to represent the whole process of risk analysis and risk evaluation.

Risk assessment runs through the safety business chain, which consists of enterprises and governments. During the risk assessment process, especially for quantitative risk assessment, risk measures play an important role in providing information on the size and level of risks [5]. Compared to qualitative risk analysis methods, the quantitative ones have the advantage of being more precise and clearer, providing a basis for objective and rational decision-making regarding risks [6], and with the continuous improvement of data computing capabilities, their disadvantage of enduring heavy calculations will gradually disappear. Due to differences in the roles and functions of safety management, enterprises and governments have different requirements for risk measures. Specifically, enterprises are often concerned about risks that describe concrete hazards in the working area. After evaluating the risk level of each hazard, those at higher levels are prioritized for stronger control. Instead of treating individuals solely as management objects, for supervision convenience, governments care more about risks that measure the overall safety of a group of hazards in the jurisdiction. This regional risk makes it easy for safety supervisors to scientifically determine regulatory direction and reasonably allocate regulatory resources. Based on the analysis, risk can be mainly categorized into two types, micro risk and macro risk, depending on the number of hazards measured. In this respect, if we measure the risk of one hazard, such as a boiler in a plant, micro risk can be utilized to identify, analyze, quantify the risk factors (factors including likelihood and consequences) based on physical and/or chemical changes that may cause an accident, and finally obtain the risk level [7]. The obtained risk can only describe the safety of that boiler itself. In contrast, if we decide to evaluate the risk level of a group of congeneric hazards in a certain area, such as all boilers in China, a micro-risk strategy would no longer be appropriate and would be replaced by macro risk, which is our focus in this study. In brief, here micro risk indicates the risk of a certain hazard. It is helpful for safety managers in enterprises to grasp the risk of each hazard and control it in a targeted manner. Meanwhile, macro risk means the risk of multiple hazards that are congeneric in a certain area. It is more suitable for safety supervisors in governments to master the overall safety level and make reasonable decisions.

Many indicators can be used to measure macro risk: However, due to the various definitions of risk [8,9,10,11,12], they are not equally significant. In general, risk consists of two elements: likelihood and consequences of an accident (or a hazardous event). Consequences cover various losses, including loss of life and economic and environmental damage [6]. However, because of different needs and purposes, some indicators only consider likelihood as risk [9,11], such as individual risk (IR) [13] and aggregated weighted risk (AWR) [14], which characterize the probability of dying due to an accident caused by a type of hazard in a certain location and within an entire area, respectively [6]. Some indicators only consider consequences as risk [8,12], such as the number of people at risk (PAR) [15], which shows the number of persons in an area that has potential disasters [6]. Some indicators only consider a single loss as a consequence: The expected value of the number of fatalities (E(N)) [16] focuses on loss of life, the curve displaying the probability of exceedance as a function of the economic damage (FD curve) [17] focuses on economic loss, and the curve showing the probability of exceedance of the time needed by the ecosystem to recover from the damage (FT curve) [18] focuses on environmental loss. In the absence of direct economic valuation of human life, the dimensional normalization of various types of loss is a non-negligible challenge, preventing the study of a risk measure that simultaneously considers likelihood and consequences, covering multiple types of losses. In this context, this work aims to solve this problem for comprehensive and scientific risk quantification.

Hazardous installations are the objects of study in this article because they are the most common and dangerous hazards in the industrial safety field, especially in China. In recent years, risk-based strategies for safety regulation have gradually become clearer and have been included in the requirements of Chinese government documents, as shown in Table 1. Under such circumstances, a mass of accident data is saved and collected to gather statistics for analyzing accident characteristics and preventing major and extraordinarily serious accidents. This is because the safety risk of a type of hazard is closely related to its accident causation. However, due to a wide range and large volume of regulatory business in China, a comprehensive and universal way of helping to perform safety supervision is urgently needed. Thus, a critical question we should find the solution to is, “How can a simple, practical, and versatile approach be established that can give full play to the advantages of risk management to achieve effective and scientific safety supervision?” To elucidate this question so as to advance safety development in China, we propose a calculation method for macro risk that simultaneously considers the likelihood and consequences of various losses [10] to measure the overall safety of hazardous industrial installations. This versatile method can help safety supervisors with goal setting and decision-making, while effectively optimizing regulatory efficiency and costs. This article is organized as follows. In Section 2, we define macro risk and report the calculation idea. A suitable metric, i.e., person-years, is introduced to normalize the dimensions of various losses. In Section 3, the process of dimensional normalization is shown in detail, and the method is discussed. In Section 4 and Section 5, two main applications are discussed and illustrated to prove the feasibility and practicality of the method. Section 6 concludes this work.

## 2. Theory and Methodology

### 2.1. Macro Risk

Risk-based strategy is one symbolic achievement in the field of insurance research [24], which has proven to have and has shown a strong connection to the safety management of industrial accidents [25]. The study of risk and risk management began in the financial field with the aim of providing a means of identifying, assessing, and mitigating unfavorable economic factors, thereby reducing the losses of companies [26]. Due to fine efficacy, the application scope and objects of risk/risk management continued to expand. Pure risk/risk management [27] thus arose, which excluded corporate financial risk. In this category, various risks, such as natural risk [28,29] related to natural disasters and technological risk [30] related to technical systems, have always been hot research areas in China in recent years. Comparatively, natural risk indicates the risk caused by hazards in nature (i.e., earthquakes, volcanic eruptions, landslides, tsunamis, and other unexpected disasters) that could endanger human life and property, while technological risk focuses on describing the risk posed by technological advances, especially the huge risk brought by large industrial systems (chemical plants, dams, production platforms, hazardous industrial installations, etc.) in human life. Moreover, in order to measure natural risk, it is often necessary to consider additional vulnerability factors [28] other than traditional possibility and severity factors. In this paper, we only concentrate on studying technological risk. Risk caused by industrial systems is the main content of technological risk [31], is important in an examination of the safety of hazards of an industrial field, and can effectively help enterprises/government implement safety management/supervision. Thus, this is usually called safety risk in China.

There is no uniform definition of safety risk. However, as similarly stated in many international standards [3,32], national standards [33,34], and research [10,35,36], safety risk is defined in this work as the combination of the likelihood of an accident (or a hazardous event) occurring and its consequences. From this classical definition, two key factors, likelihood and consequences, have dominated the size of risk. To quantify risk, a mathematical function is therefore often raised and utilized [6,37]:

(1) R=P·L,

where *R* represents risk, *P* indicates the probability that an accident will occur [34], and *L* denotes the loss resulting from an accident [35].

Based on the theory above, macro risk is further proposed to measure the overall safety of a type of hazard, i.e., the collective risk of hazards that come from a homogeneous group in a certain area and period (usually for a year). Although the risk of each congeneric hazard is not exactly the same due to exposure to various external environments, macro risk is considered to be the whole regional risk level through characterization of the average risk of all individuals in an area, which is a sufficient interpretation of the connotation of “macro”. In other words, the size of macro risk is determined not by the number of hazards in an area but by the average level of risk that each individual has reached.

From the above concept, to quantitatively study the macro risk of hazardous industrial installations is to seek a reasonable calculation method for average risk. In this article, we try to solve this problem via systematic accident statistics and analysis, because complete historical data can accurately and expediently reflect accident laws and hazard characteristics. An implementation of the method roughly follows the three steps below:

- With total risk unchanged in an area, assume that all congeneric installations are identical and that each individual has the same risk. Under this assumption, the same risk is thus the required average risk, namely
  (2) $$\sum_{i=1}^{N}R_{i}=N\bar{R},$$
  where *N* represents the number of installations in a certain area, *R_i_* indicates the risk of the *i*th installation, and $\bar{R}$ denotes the average risk;
- How is it ensured that total risk is unchanged? A simple way is to utilize the historical accident data (annual data) of the installations to calculate average risk. This can be used because risk is closely related to accident probability and consequences (Equation (1));
- Calculate the average risk based on probability theory. According to the two steps above, the problem is then transformed into an independent repetitive trial for an individual. Combining Equation (1) with related probability theory [38], we can finally obtain an expression for calculating the macro risk, as follows:
  (3) $$R_{m}=\bar{R}=p\cdot E\left(L\right)=\frac{M}{N}\cdot \frac{1}{M}\cdot \sum_{j=1}^{M}L_{j}=\frac{1}{N}L_{t},$$
  where *R_m_* represents the macro risk of a certain type of installation, *p* indicates the frequency of accidents per year, *E*(*L*) denotes the expected value of *L* per year, *M* is the number of installation-related accidents per year in the area, *L_j_* is the loss of the *j*th accident, and *L_t_* is the total loss of accidents per year in the area.

However, due to the various components of loss (e.g., death, injury, economic damage), the value of *L_t_* cannot be obtained directly. Therefore, a dimensional normalization of the various losses is urgently needed.

### 2.2. Loss Equivalent

Based on representative studies on quantitative risk assessments [6,37,39,40], the consequence of an accident is roughly divided into the following three categories: loss of life, economic loss, and environmental damage. Because environmental damage can also be expressed in monetary terms [6,40,41], in this study, we just focus on a consequence that mainly includes loss of life and economic loss (as follows), which also agrees well with statistical data from the Chinese government [42]:

(4) $$L_{t}=\left\{L_{h},\;L_{e}\right\},$$

where *L_h_* represents the loss of life associated with the accidents, which includes deaths, serious injuries, and slight injuries; and *L_e_* represents economic loss, which includes direct economic loss and indirect economic loss [43,44]. The different dimensions of *L_h_* and *L_e_* make them inoperable while calculating *L_t_*. Additionally, in the view of many people, it is unethical to directly convert human life into a monetary value [6]. Fortunately, we found an extremely suitable metric, “person-year”, to help achieve dimension normalization.

**Figure 1:**
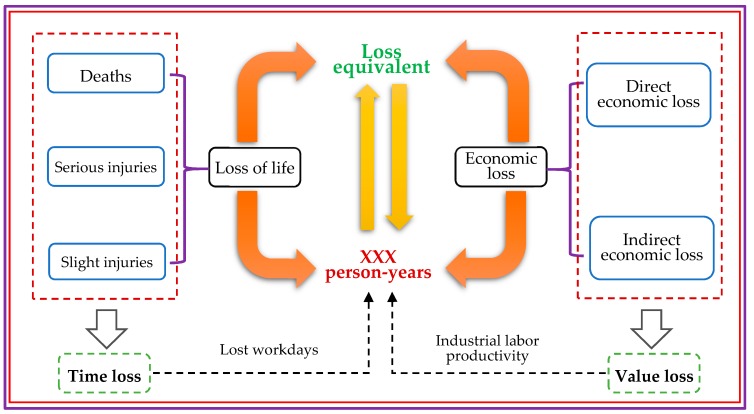
Schematic diagram of the dimensional normalization process of various losses.

A “person-year” is a compound unit whose number is often used to calculate birth rate, mortality, and other indicators in the field of demographic statistics [45]. It is composed of the product of the number of people and the length of time. The meaning of “person-year” is easy to understand: For instance, one person-year indicates that one person survived for one year, and two person-years indicates that either two people survived for one year or one person survived for two years. To normalize different dimensions, the number of person-years will be introduced and used as a loss equivalent to set up a method of conversion between the loss of life and economic loss. A schematic diagram can be illustrated as follows. As shown in Figure 1, the components of loss of life are considered to be time losses, which is converted into person-years by calculating lost workdays. Comparatively, economic loss can be treated as value loss so that it can be converted into person-years using industrial labor productivity. Although the methods of conversion are different, their connotation remains consistent and will be explained in detail in Section 3.

## 3. Dimensional Normalization of Varied Losses

### 3.1. Loss of Life

People create wealth for family and society through their time. The nature of human loss of life, especially for workers, is actually a loss of work time [46]. A lost workday is a well-defined value and can serve as a good surrogate for severity [47]. Thus, loss of life can be measured from the estimated number of lost workdays [48,49,50]. For instance, according to Chinese standards [46,51], work-related employee injuries of different severities are assigned different lost workdays on the basis of a percentage of total permanent disabilities [48]. Among them, death is equivalent to 6000 lost workdays, while slight injuries and serious injuries are partitioned based on a threshold of 105 lost workdays. If the injury statistics are sufficient, the number of lost workdays corresponding to disabilities can just be summed. Calculated using the binomial distribution of disability statistics, the expected values of a slight injury and a serious injury can also be determined as 100 and 3250 lost workdays, respectively, for use when historical accident data cannot provide detailed injury information.

After loss of life is equivalently converted into the number of lost workdays, another process designed to further change the number of lost workdays into person-years should be performed. Here, based on the object of the study, i.e., industrial installations, we again optimize and define one person-year, which can be considered one person working for one year. Obviously, there are approximately 250 workdays for one person in one year. Therefore, the expression converting loss of life to person-years can be approximately defined as follows:

(5) $$L_{h}^{*}=\frac{l_{d}\cdot S_{d}+l_{s}\cdot S_{s}+l_{r}\cdot S_{r}}{250},$$

where L_h_^*^ represents the person-years of *L_h_*, which is used to obtain *L_t_*; *l_d_* indicates the number of deaths in an accident; *l_s_* indicates the number of slight injuries; *l_r_* indicates the number of serious injuries; *S_d_* denotes the expected value of lost workdays corresponding to a death; *S_s_* denotes the expected value of lost workdays corresponding to a slight injury; and *S_r_* denotes the expected value of lost workdays corresponding to a serious injury.

### 3.2. Economic Loss

In the previous section, using time-workdays, we introduced the process of converting loss of life into person-years. Meanwhile, as stated above, people create wealth during their working life. In other words, people create value by consuming time. Based on this argument, person-years based on time can be equated to person-years based on value in the same period. Thus, to convert economic loss to person-years, we introduce an annual economic indicator called industrial labor productivity. Industrial labor productivity can be defined as follows:

(6) $$ILP=\frac{IAV}{Q},$$

where *ILP* represents industrial labor productivity, which is used to measure the ability of one person to create wealth for society per year in a certain area; *IAV* indicates the industrial added value per year, which is a major component of gross domestic product (GDP) and describes the total value created by industrial production activities; and *Q* denotes the number of employees. *ILP* reflects the value generated by one person-year. To derive person-years based on value from the economic loss of accidents, the calculation method can be defined as follows:

(7) $$L_{e}^{*}=\frac{l_{a}+l_{b}}{ILP}=\frac{l_{a}+\omega l_{a}}{ILP},$$

where L_e_^*^ represents the person-years of *L_e_*, which is also used to obtain *L_t_*; *l_a_* indicates the direct economic loss of an accident; *l_b_* indicates the indirect economic loss of an accident; and ω denotes the value of a ratio of indirect to direct loss [52], which varies significantly from 0.75:1 [53] to 20:1 [54] or more, depending on the region [55,56,57] and industry [44,58].

Based on all of the analysis and inference above, we can finally conclude the expression for calculating the macro risk of hazardous industrial installations as follows:

(8) $$R_{m}=\frac{1}{N}L_{t}=\frac{1}{N}\left(L_{h}^{*}+L_{e}^{*}\right)=\frac{1}{N}\left(\frac{l_{d}\cdot S_{d}+l_{s}\cdot S_{s}+l_{r}\cdot S_{r}}{250}+\frac{l_{a}+\omega l_{a}}{ILP}\right).$$

Compared to the aforementioned international existing tools used for macro risk evaluation, such as those (IR, AWR) only considering likelihood as risk, that (PAR) only considering consequences as risk, and those (E(N), FD curve, and FT curve) only considering single loss as a consequence, the proposed method simultaneously takes the likelihood and consequences of an accident into account and successfully achieves the dimensional normalization of various losses via introducing person-years (in the case of avoiding direct economic valuation of human life).

## 4. Discussion

The core data used in this study comes from accident statistics. Therefore, current macro risk is usually obtained based on data from previous years. In other words, the value of macro risk has a chronergy. For safety supervisors, the chronergy can effectively help with goal setting and decision-making because supervisors need to calculate the macro risk at the end of every year or at the beginning of the next year to fully understand the safety status of hazardous installations and establish a targeted regulatory plan with achievable goals of risk reduction according to results.

Different from concrete installation risk, macro risk focuses on annotating the overall risk level of entire congeneric installations in an area. Instead of calculating the risk of all individuals in aggregate to obtain total risk, we just use average risk, which means the number of hazards does not dominate the size of the macro risk. In this context, supervisors can grasp the risk level in a single area and compare it to various jurisdictions, so as to reasonably allocate regulatory resources. In addition to comparing the macro risk of congeneric installations in different areas, it is also feasible to compare different types of hazards in one area. Because of this, supervisors can discover the laws between different risks and determine the main regulatory direction. To demonstrate the discussion above, two examples of applications from China are provided in the next section.

Without exception, the limitations of the model proposed should also be pointed out. Due to the use of historical accident statistics, the method is suitable for evaluating the risk level at the current time stage (usually five years, the term of the National People’s Congress in China) rather than predicting its future trends. However, the obtained results can be used as annual risk evaluation criteria for the next period. Additionally, the model should be applied to large-scale enterprises or governments to ensure sufficient enough data to get accurate results.

## 5. Practical Applications

### 5.1. Macro Risk of Boilers in China

In China, boilers are considered “special equipment” that pose a substantial risk to human safety [59]. Risk measures have become vital to safety supervision throughout the lifecycles of special equipment. In this context, we intend to take boilers as an example to study macro risk. As an example, accident statistics from 2006 to 2011 in China [42,60,61,62,63,64] were selected as the data source for calculation, as shown in Table 2.

As previously mentioned, using data from 2011, the macro risk in 2012 (3.16 × 10^−3^) could be determined according to Equation (8), where *S_d_* and *S_r_* were 6000 and 3250, respectively [33,38], and the value of ω was 8.5: This was based on a study by Wang et al. [65], who systematically studied the ratio of indirect to direct loss from major and extraordinarily serious industrial accidents in China. To briefly judge whether this macro risk reached an acceptable level, the average macro risk from the previous five years (from 2006 to 2010) could be treated as a risk acceptance criterion. In this way, the criterion became more and more stringent as time passed [66], which helped continuously reduce risk. If macro risk is lower than average macro risk, the risk is considered acceptable. In contrast, if macro risk is higher than average macro risk, the risk may be deemed unacceptable and may require control. Under this circumstance, supervisors must improve regulatory intensity for risk reduction. Here, the macro risk in 2012 (3.16 × 10^−3^) was higher than the average macro risk (3.11 × 10^−3^), indicating that the new annual regulatory plan should have included an item that was likely to apply stronger supervision to boilers.

For supervisors, in addition to fully understanding and mastering the macro risk of boilers in the whole country to comprehensively plan and set goals, a risk level comparison in various jurisdictions is also necessary to reasonably allocate regulatory resources. As further explanation, we assumed that the macro risk of boilers in 2012 was 3.3 × 10^−3^ in Beijing after calculation and 2.9 × 10^−3^ in Shanghai. The risk level in Beijing was clearly much higher than in Shanghai, and it even exceeded average macro risk (3.11 × 10^−3^). Thus, more regulatory resources should have been allocated to Beijing, while Shanghai did not need as many. Only in this way can regulatory costs be greatly reduced.

### 5.2. Absolute Risk and Relative Risk

Due to the universality of the proposed method, another application focuses on determining which type of hazardous installations should be given preference for full supervision. To illustrate this, we took boilers and another type of special equipment, hoisting machinery, as an example. The related data [50,51,52,53,54,55,56,57,58,59,60,61,62,63,64] for calculation are shown in Table 3.

To disclose the rules of accidents hidden in statistical data and avoid contingency, an analysis of these two types of installations was performed based on five-year data using the proposed method. By averaging the macro risks from 2006 to 2010, results were determined for boilers (3.11 × 10^−3^) and hoisting machinery (5.32 × 10^−3^). The risk level of hoisting machineries was much higher than that of boilers, indicating that hoisting machinery required more comprehensive supervision than boilers did. In other words, for safety supervisors, if both types of installations need to be controlled at the same time, hoisting machinery is of a higher priority.

**Figure 2:**
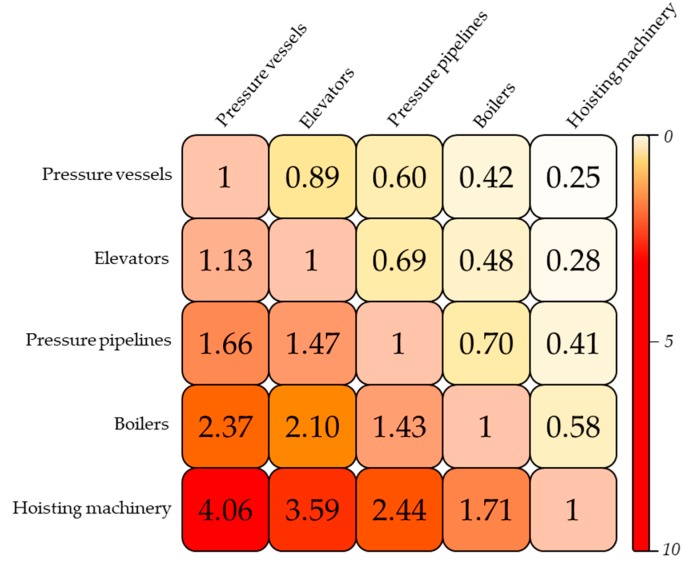
Relative risk matrix of multiple types of hazardous industrial installations in China in the period from 2006 to 2010.

The macro risk comparison of various types of installations provided data and technical support for determining regulatory direction. To make the process more convenient, we could utilize absolute risk and relative risk indicators. Absolute risk was set up to represent the macro risk itself, while relative risk was the ratio of various macro risks differentiated by installation type. Again, using boilers and hoisting machinery as an example, relative risk could be defined as follows:

(9) $$R_{\mathrm{hoisting}\;\mathrm{machinery}-\mathrm{boiler}}^{*}=\frac{\bar{R_{m\left(\mathrm{hoisting}\;\mathrm{machinery}\right)}}}{\bar{R_{m\left(\mathrm{boiler}\right)}}},$$

where R^*^_hoisting machinery–boiler_ represents the boiler-based relative risk of hoisting machinery; $\bar{R_{m\left(\mathrm{hoisting}\;\mathrm{machinery}\right)}}$ indicates the average macro risk of hoisting machinery; and $\bar{R_{m\left(\mathrm{boiler}\right)}}$ denotes the average macro risk of boilers. By substituting the macro risks of boilers (3.11 × 10^−3^) and hoisting the machinery (5.32 × 10^−3^) obtained above into Equation (9), R^*^_hoisting machinery–boiler_ (~1.71) was determined. Based on this result, it could be understood that the risk of one hoisting machine in China was equivalent to that of 1.71 boilers. Further, also taking accident statistics from the period 2006 to 2010 as an example, we obtained the relative risk of various hazardous industrial installations based on a certain macro risk, as shown in Figure 2 (here, also based on the study by Wang et al. [65], we considered ω to be an unchanged constant, 8.5, in these high-danger installation-enabled accidents, which was less affected by the type of industry).

According to this line of thought in the analysis, when simultaneous supervision is needed for multiple installations, set one type of installation with an intermediate macro risk as a benchmark, and the risk levels of other types of installations are cleared at once. Hence, supervisors can make decisions on which types of installations should be prioritized and focused on based on the laws and characteristics of risks.

## 6. Conclusions

To measure the overall safety of hazardous industrial installations, we proposed a calculation method for macro risk that describes the risk level of a type of hazard in a certain area. Different from the risk of a concrete installation, macro risk focuses on annotating the overall risk of all congeneric installations in an area. Thus, in our method, accident statistics and analysis become the main means of accurately and sufficiently reflecting the accident laws and characteristics of hazards. During the dimensional normalization of various losses, we introduced a suitable method (utilizing person-years) for converting loss of life and economic loss into person-years, which helped achieve the model. To further prove the feasibility of the proposed method, we demonstrated two main applications. Safety supervisors, by using this calculation method, in addition to fully understanding and mastering the macro risk of the whole country area for comprehensive planning and goal setting, could also perform a comparison of the risk level in various jurisdictions for reasonable regulatory resource allocation. When the simultaneous supervision of multiple installations is required, a comparison of the macro risks of various types of installations could help to determine the regulatory direction. In addition, it remains necessary to enhance the study of indirect economic loss in our future study. Statistics on and the measurement of indirect economic loss in an accident are not easy and are thus not reflected in the Chinese government’s public data. If an accurate value of indirect economic loss could be acquired, it would inevitably promote the study and application of macro risk in China. However, when there is no accurate indirect economic loss, the estimated value can also be used via the ratio of indirect to direct loss (ω), which has always been studied by researchers. In this paper, we just roughly considered ω as a constant for demonstrating the possible applications of macro risk. We believe that more detailed and scientific guidance for ω should be included in our future work to meet different accident types and scenarios. Finally, it should be noted that although the proposed method is based on national conditions in China, its design philosophy and methodology are internationally universal, supporting the globalization of macro risk.

## Figures and Tables

**Table 1:** Representative Chinese government documents related to risk management and the information within from five recent years.

Year	Names	Agencies That Issue Documents	Key Points on Risk Management
2016	Guidelines for curbing major and extraordinarily serious accidents [19]	The State Council of China	Sound safety risk assessment standards; Comprehensively identify and evaluate safety risk levels; Establish a safety risk ranking, management, and control system;
2016	Suggestions on building a dual prevention mechanism for the implementation of guidelines for curbing major and extraordinarily serious accidents [20]	The State Council of China	Build a dual prevention mechanism, including risk management and “*Yinhuan*” (hidden dangers) check and control; Scientifically evaluate risk levels, effectively manage regional risks;
2016	Opinions on promoting reform and development in the safe production field [21]	Communist Party of China Central Committee, the State Council of China	Enhance risk assessment and warning for key industries, regions, and enterprises;
2017	The 13th five-year plan on safe production [22]	The State Council of China	Promote the establishment of a risk management system in various fields, including coal mining and the chemical industry; Improve the capabilities of risk prevention and control of enterprises;
2017	Notice on further strengthening the safety production of central enterprises [23]	China’s State Administration of Work Safety	Strengthen safe production via source treatment and risk prevention and control;

**Table 2:** Statistics on boiler-related accidents and industrial labor productivity (*ILP)* in China from 2006 to 2011.

Year	*N*	*l_d_*	*l_r_* ^1^	*l_a_* (Chinese Yuan (CNY))	*ILP* (CNY)
2011	62.03 × 10^4^	24	54	723.3 × 10^4^	10.07 × 10^4^
2010	60.73 × 10^4^	24	38	649.3 × 10^4^	8.77 × 10^4^
2009	59.52 × 10^4^	23	64	566.35 × 10^4^	7.60 × 10^4^
2008	57.82 × 10^4^	24	60	515.23 × 10^4^	7.30 × 10^4^
2007	53.41 × 10^4^	19	35	66.3 × 10^4^	6.27 × 10^4^
2006	54.30 × 10^4^	20	60	446.2 × 10^4^	5.52 × 10^4^

^1^ Since the Chinese government only records unclassified injuries and the ratio of injuries to deaths, the statistics are always lower than the actual level. All injuries were considered to be serious injuries (*l_r_*) in Section 5.

**Table 3:** Statistics on accidents related to hoisting machinery in China from 2006 to 2010.

Year	*N*	*l_d_*	*l_r_*	*l_a_* (CNY)
2010	150 × 10^4^	83	27	2839.78 × 10^4^
2009	135.27 × 10^4^	85	42	1786.68 × 10^4^
2008	118.28 × 10^4^	74	31	7085.87 × 10^4^
2007	95.79 × 10^4^	94	33	1267.4 × 10^4^
2006	82.36 × 10^4^	80	35	1001.3 × 10^4^
